# Nonhuman Primate Models Used to Study Pelvic Inflammatory Disease Caused by *Chlamydia trachomatis*

**DOI:** 10.1155/2011/675360

**Published:** 2011-08-17

**Authors:** Jason D. Bell, Ingrid L. Bergin, Kelsey Schmidt, Melissa K. Zochowski, David M. Aronoff, Dorothy L. Patton

**Affiliations:** ^1^Department of Obstetrics & Gynecology, University of Michigan, 1500 East Medical Center Drive, L4510, Ann Arbor, MI 48109, USA; ^2^Unit for Laboratory Animal Medicine, University of Michigan, Ann Arbor, MI 48109, USA; ^3^College of Literature, Science, and the Arts, University of Michigan, Ann Arbor, MI 48109, USA; ^4^Reproductive Sciences Program and the Division of Infectious Diseases, Department of Internal Medicine, Department of Microbiology & Immunology, University of Michigan, Ann Arbor, MI 48109, USA; ^5^Department of Obstetrics & Gynecology, University of Washington, Seattle, WA 98195, USA

## Abstract

Pelvic inflammatory disease (PID) is a global health concern that is associated with significant morbidity and is a major cause of infertility. Throughout history animals have been used for anatomical studies and later as models of human disease. In particular, nonhuman primates (NHPs) have permitted investigations of human disease in a biologically, physiologically, and anatomically similar system. The use of NHPs as human PID models has led to a greater understanding of the primary microorganisms that cause disease (e.g., *Chlamydia trachomatis and Neisseria gonorroheae*), the pathogenesis of infection and its complications, and the treatment of people with PID. This paper explores historical and contemporary aspects of NHP modeling of chlamydial PID, with an emphasis on advantages and limitations of this approach and future directions for this research.

## 1. Introduction

The Center for Disease Control and Prevention approximates that 750,000 women in the United States contract pelvic inflammatory disease (PID) each year and 10% of these cases result in infertility [1]. It is the ascent of bacterial organisms to the upper reproductive tract that causes PID. The majority of PID cases are due to sexually transmitted infections with *Chlamydia trachomatis *and *Neisseria gonorroheae*. *C. trachomatis *is a common cause of PID in women and is usually an asymptomatic infection, which allows the organism to remain undetected as it ascends into the upper reproductive tract [2]. High rates of infertility and morbidity resulting from PID are a global concern [3]. 

Nonhuman primates (NHPs) have a long history of use in biomedical research. In the first century A.D., Barbary apes (*Macaca sylvan*) were likely the first primates used in research [4]. These apes were used as replacements for human cadavers in anatomical studies. In the late 19th century, recognition of the similarities between humans and NHPs promoted the usefulness of these animals in research [5, 6]. In the last hundred years the primate has become a common research subject in many United States based laboratories. As this interest evolved, more primate species were used in biomedical research. Originally, primates were used strictly as anatomical models, but this evolved to include studies of disease pathogenesis and the testing of novel medical technologies. The similarities between the anatomy and physiology of NHPs and humans have made such modeling successful [7–9]. The specific utilization of NHPs in reproductive studies has occurred more recently and continues to expand. 

In 1950, Te Linde and Scott noted the effectiveness of using NHPs (rhesus monkey) in the study of endometriosis [10]. The anatomical and physiologic similarities of NHPs permitted the modeling of diseases that would otherwise be impossible, or unethical, in humans. Because no single species of NHP entirely reproduces the pathophysiology of human disease, various NHPs (marmoset, macaque, grivet, and baboon) have been used. NHP models of sexually transmitted infections have permitted a better understanding of disease pathogenesis, the effectiveness of different treatment modalities and have accelerated renewed research interests in prevention strategies, including topical microbicide and vaccine development. NHP models of PID, particularly *C. trachomatis*-associated PID, have been used for over five decades. *C. trachomatis *has been used to successfully infect a variety of NHPs: chimpanzees, marmosets, grivets, macaques, and baboons [11–15]. Primates provide an attractive model for PID because, unlike other animal models (sheep, guinea pigs, pigs, and mice), NHP reproductive tracts are of a similar shape and size to those of women [16]. What is more, contemporary NHP PID models employ the use of human serovars of *C. trachomatis *to induce PID, as they are naturally susceptible to infection of the lower and upper genital tract after challenge. When smaller animal models are used nonhuman strains, exogenous pretreatment with hormones as well as use of PID-inducing pathogens often need to be employed because human-derived bacterial strains may not cause a similar disease in the particular animal [17, 18]. This paper presents the current status of NHP models for the study of experimentally induced chlamydial PID.

## 2. The Rationale for NHP Use in Biomedical Research

In theory, the best model for human disease is the human; however ethical and practical concerns prohibit many fundamental studies. Thus, NHPs serve a unique niche in biomedical research. The evolutionary closeness that the NHPs share with *Homo sapiens *helps ensure biologically similar models with both predictive and discriminative abilities unavailable in other animal models [19]. Though research performed in rodents, and other small animals has several advantages: lower cost, ease of handling, and the ability to genetically alter them (*e.g.,* knockout mice), the results are not always translatable to human disease. 

The use of NHPs in PID models has been able to successfully fulfill Koch's postulates to prove that *Chlamydia* causes PID [12, 14, 20, 21]. NHP models allow for detailed observations of disease progression, that is, the pathogenesis of infection, to be studied *in vivo*. Likewise, one can effectively examine host immune and physiological responses independently from studying microbial pathogenesis [22]. While these advantages are shared with other animals, a unique advantage of NHPs is their relatively long life span that permits animals to be reused in subsequent independent studies during their lifetime. In addition, many NHPs continue their menstrual cycle while in captivity, supporting their use as reproductive health models. Differences both subtle and great have led to the creation of PID models among different species (Table 1). 

Though NHPs have numerous advantages they are not without limitations. Using NHPs is costly, requires adequate facilities, veterinarian staff support, expertise, and technical staff to care for the animals. And, due to the increasing debate regarding vivisection, adequate security is imperative.

## 3. Early NHP Studies of *Chlamydia* Pathogenesis

Thygeson, an ophthalmologist, studied ocular chlamydial infections and reported that maternal-to-infant transmission induced chlamydial cervicitis (Table 2) [11]. Using material collected from the eye of an infant with inclusion blennorrhea the cervix of two baboons were directly inoculated. One animal developed a marked cervicitis with purulent material in the cervical canal at 12 days postinoculation [11]. The second animal had no response after the first inoculation. With a repeated inoculation this animal developed mild cervicitis but without a purulent discharge. Interestingly, Thygeson and his colleagues were unable to demonstrate the detection of inclusion bodies after inoculation in either animal.

Alexander and colleagues used the Taiwan monkey in a similar fashion, inoculating the cervix of pregnant monkeys with TRIC (Table 2) [31]. Like Thygeson, they reported mild erythema of the lower genital tract and were unable to detect inclusion bodies after inoculation. These early studies were not designed to model PID and had limited results; but they provided important foundation for later use of NHPs in PID modeling.

## 4. Early NHP Models of Lower Genital Tract *Chlamydia* Infection

Johnson et al. used the marmoset to show that an acute inflammatory response reaction occurred with a vaginal inoculation of *C. trachomatis* (Table 2) [20]. This response lasted 10–42 days. Acute cervicitis was characterized by erythema, occasional edema, and the presence of purulent cervical mucus [35]. A neutrophil-rich leukocyte response was shown, and chlamydial inclusions were demonstrated in the epithelium. The lower genital tract response was characterized using histology and colposcopy. Interestingly, animals with repeated infection, with either homologous or heterologous chlamydial strains, had decreased inflammatory responses, shorter durations of infection and those that had previous inoculations were able to eliminate the infection within one week [34]. 

Nearly all animals in their study developed either an IgM or IgG antibody response. Despite the number of reinfections, the highest IgM titer was 1:16 where the IgG response tended to increase with subsequent inoculations suggesting that the animals had developed an adaptive immune response. The focus of this group's work was studying the lower genital tract, but one case of acute endometritis and salpingitis was observed histologically upon autopsy. However, the authors did not have microbiologic studies of the upper tract prior to autopsy and were unable to identify intracytoplasmic chlamydial inclusions in epithelial cells.

## 5. Development of PID in NHPs

Concomitant with Johnson et al.'s work in marmosets, Moller et al. did similar work in grivet monkeys (Table 2) [46]. By directly inoculating the fallopian tubes and the endometrial cavity, grivet monkeys developed acute self-limited salpingitis. The histological findings in the tubal tissues post-inoculation (infiltration of the subepithelial and mucosal epithelium, abundant lymphocytes, and polymorphonuclear (PMN) leukocytes, and exudate in the tubal lumen with small clusters of desquamated epithelium) mirrored what is considered to be the hallmark signs of acute salpingitis in women [32]. Moller also showed that there was an activated immune response associated with inoculation with *C. trachomatis*. The antibody response was that of a primary infection with an IgM to IgG antibody seroconversion [32]. Additionally, in the animal that was infected in the endometrial cavity they documented ascent of the *C. trachomatis* to the fallopian tubes.

A later study by this same group found that direct cervical inoculation with *C. trachomatis* resulted in acute salpingitis [12]. These findings confirmed Johnson's results in the marmoset. They, too, satisfied Koch's postulates and reisolated the organism. Again, the cervical infection with *C. trachomatis *resulted in “classic” histological findings for acute salpingitis as noted in humans, and like the previous trial, the serological conversion seen post cervical inoculation was an IgM to IgG seroconversion.

## 6. Current NHP Models of PID

Using direct tubal inoculation, Patton and colleagues demonstrated a histopathologically similar acute salpingitis in the pigtailed macaque to that noted by Moller et al. in the grivet monkey (Table 2) [36, 37]. In the pigtailed macaques and grivet monkeys a single inoculation of *C. trachomatis *resulted in self-limited tubal inflammation without evidence of tubal scarring. This inflammatory period peaked at day 14 and resolved by days 28–35. However, with repeated inoculations a chronic salpingitis with extensive tubal scarring, distal tubal obstruction, and peritubal adhesions was induced [14]. 

This picture of chronic salpingitis after repeated infections led Patton and colleagues to develop a subcutaneous pocket model in cynomolgus, rhesus and pigtailed monkeys so that the tubal response to infection could be easily monitored [38]. The histopathology again showed a progression of the inflammation of the mucosal and muscular layers as well as a transition from lymphocytic cells, PMNs, and plasma cells. Like previous studies this model showed a short-lived infection that was microbiologically, immunologically, and histopathologically similar to studies in intact monkeys where inoculation had occurred directly to the fallopian tube [36]. An important advantage of the pocket model is that separate anatomic tubal sites are established and can be individually manipulated per study design. However, duration of infection was shorter. Though this model was successful in studying the kinetics of acute and chronic chlamydial infection in tubal tissues, the use of the intact NHP reproductive tract is essential to investigate (scarring and fibrosis) pathogenesis of *Chlamydia*-induced PID.

Recognizing that the intact NHP model provided a more analogous model of human disease Patton and colleagues investigated how infection ascends from the lower into the upper reproductive tract. Repeated weekly (×five weeks) cervical inoculations in four pig-tailed macaques caused peritubal adhesions in all four monkeys. In contrast, in a separate study, after primary cervical inoculation, seven animals were rechallenged at the cervix only after the cervix had become culturally negative for two consecutive weeks; none developed adhesions [39]. 

With this new model of upper tract infection Patton et al. had a system that could be used to evaluate cellular immunity associated with chlamydial infections and chronic immune responses to infection that are associated with the tubal damage. Additionally, in another study it was demonstrated with repeated inoculations (after cessation of infection) animals had a shorter duration of infection [40]. With this protective immune response there was no correlation between the antibody titers (IgG) at the time of reinoculation and the success of reinoculation. Attempting to further define the immune response associated with chlamydial infection Patton et al. used their subcutaneous pocket model to better understand this association [41]. They showed the same histological changes in the tubal tissues as seen in their previous work; however, after resolution with primary infection several pockets were tested by injection of rhsp60 (recombinant heat-shock protein 60) or sham injection. Those pockets that were injected with rhsp60 showed a marked increase in inflammation at 24 hours and an even greater response at 48 hours. After 48 hours the cellular infiltrate consisted primarily of mononuclear lymphocytes that permeated the submucosal tissues, which is consistent with a delayed hypersensitivity response [41]. Later, again using the subcutaneous pocket model as well as cervical/tubal inoculations mRNAs for IFN-*γ*, IL-2, IL-6, IL-10, but not IL-4, were isolated from salpingeal tissues [42]. IFN-*γ* and IL-2 are made by TH1 CD4 T cells, whereas TH2 cells make IL-4. IL-6 and IL-10 are made by both TH1 and TH2 cells. These results suggested that TH1-like cytokines are made by repeated infection with *C. trachomatis *[42]. 

Recently, Patton et al. have used their model of PID to evaluate different antibiotic treatments for chlamydial infections [43]. In this study different treatment options (doxycycline, azithromycin) were tested versus placebo (untreated infection). As expected, animals that were in the placebo arm developed PID. However, in the two treatment arms there were differences. Those animals treated with doxycycline developed inflammatory cell profiles similar to those of the untreated animals. In contrast the azithromycin-treated animals had significantly reduced levels of inflammatory infiltrates. Azithromycin treatment ameliorated the immune response and was highly effective in eradicating *C. trachomatis* from the lower and upper reproductive tract tissues [43]. 

As a vaccine for *C. trachomatis *has been not yet been developed, recent studies have focused on novel ways to prevent infection. Female-controlled products, such as microbicides, provide a critical opportunity to prevent sexually transmitted infections. In 1999, Patton et al. were awarded a Sexually transmitted Disease Prevention-Primate Contract designed to comparatively assess the safety of topical microbicide products to cervical and vaginal tissues after repeated exposure and to evaluate their efficacy in preventing cervical chlamydial infection. Over 28 products have been evaluated (gels, films, capsules, etc.) for efficacy, safety and 9 for prevention of *C. trachomatis *infection [44]. Four of the candidate products tested were associated with tissue abnormalities that included epithelial friability, abrasion, and disruption [44]. These results demonstrated early warnings of products with potentially deleterious cervicovaginal effects, which lead to reformulation of several products [44]. In the rectal safety and efficacy studies, 12 products have been evaluated for safety and one was additionally tested for efficacy against rectal chlamydial infection. Two products had an unacceptable safety profile, and no protection was observed in prevention of acquisition of rectal chlamydial infection [45]. 

Until recently, the baboon had not been used in PID models. It is probable that the size, strength, temperament, and space required to use these animals have limited their use. However, next to the great apes (chimpanzee, gorilla, orangutan), the baboon is the animal most similar to humans in reproductive anatomy, physiology, and biochemistry, including hormonal fluctuations and cycling [47]. Another benefit of the baboon is that they have a straight (rectilinear) cervical canal, not tortuous like the chimpanzee and macaque, which permits transcervical procedures (i.e., endometrial sampling, intrauterine contraception placement) in a manner that mirrors practice in women [8]. Given this similarity to humans, the baboon has been used in vaccine trials for *C. trachomatis *[48]. As mentioned above one of the earliest trials to use *C. trachomatis* in baboons was in the 1930s by Thygeson and Mengert, [11]. Those trials showed that the baboon could become infected with *C. trachomatis* and develop cervicitis. Furthermore, work by Digiacomo and colleagues showed that the genital tract of male baboons could be infected and continue to shed chlamydial organisms for approximately 3 months after initial inoculation [49].

Based on the studies previously conducted in the baboon, as well as the Patton model of PID in the macaque, Bell et al. recently developed a new model of PID in baboons (Table 2) [21]. This work was conducted in wild-caught olive baboons, and *C. trachomatis*was used as the infectious agent. Bell et al. showed that the baboon was highly susceptible to *C. trachomatis. *Moreover, that with even a single inoculation, the organism ascended to the upper reproductive tract, as both Johnson and Moller had shown in their models [21]. Bacteria caused changes consistent with infection in the upper reproductive tract. Like women with *C. trachomatis* infections, the animals in this trial developed varying degrees of infection, from mild cervicitis to PID. Furthermore, Bell and colleagues demonstrated that multiple inoculations of *C. trachomatis* were able to drive the infection into the upper reproductive tract (unpublished data), and thus, like the work of Patton, have established a working model of PID.

## 7. Discussion

Each of the models of PID above offers investigators the ability to test scientific questions in animals that are anatomically, biologically, and physiologically similar to humans. Although there are a number of animal models, each model reflects some aspect(s) of human disease and appropriate models that should be utilized to study the desired outcome. Interestingly, inoculation with *C. trachomatis* in NHPs does not lead to infection in all animals, with some animals even failing to show cervicitis. This observation further supports the similarity to variable clinical picture that is seen in women, where some women remain asymptomatic. This point serves to further strengthen the similarity between the NHP model of PID and the human disease state. The basis for this heterogeneity requires further attention.

## 8. Conclusions

Research concerning PID has progressed greatly in the past century and continues to advance each year. The use of NHPs has allowed and will continue to allow for a greater understanding of the disease. Through the ethical and humane use of NHP models, the field of reproductive infectious disease has continued to improve our scientific understanding of the disease and has helped to improve treatment measures. The continued use of these models will allow for investigators to answer questions about human disease that cannot be easily studied in humans.

## Figures and Tables

**Table 1:** List of NHP used in reproductive health research.

Animal	Species	Average Life span (years)	Average size (cm)	Average weight (kg)	Menstrual length (days)*	Advantages/disadvantages
Olive baboon	*Papio anubis*	25–30	M: 70 F: 60	M: 24 F: 14.7	30–35 [23]	Advantages: anatomical and physiological closeness to humans, large size (facilitates procedures), straight cervix, readily available, non-threatened, can visually monitor cycle stage with greatest precision Disadvantages: large size and strength, large housing requirements
African Green Monkey (Vervet, Grivet) [24, 25]	*Chlorocebus aethiops* (formerly *Cercopithecus aethiops) *	11–13 (captive)	M: 49 F: 42.6	M: 5.5 F: 4.1	30–32 [26]	Advantages: anatomical and physiological closeness to humans, manageable size, readily available, non-threatened Disadvantages: tortuous Cervix, cannot visually monitor cycle stage
Common marmoset	*Callithrix jacchus*	12 (wild)	M: 18.8 F: 18.5	.350–.450* [27]	22–28	Advantages: anatomical and physiological closeness to humans, manageable size, readily available, non-threatened Disadvantages: no menstruation (has estrus cycle) [4], small size (precludes certain procedures); ovulation pattern slightly different from humans (twinning is the normal state), cannot visually monitor cycle stage

Macaques						
Pigtailed	*Macaca nemestrina*	26	M: 49.5–56.4 F: 46.7–56.4	M: 6.2–14.5 F: 4.7–10.9	30–40 [28]	Advantages: anatomy of reproductive tract tissues and physiology of menstrual cycle similar to humans, size (facilitates most procedures), non-threatened, can visually monitor cycle stage to some degree Disadvantages: limited availability, some species are seasonal breeders (rhesus, possibly Formosan rock macaque), tortuous cervix, aggressive temperament (rhesus), zoonotic disease risk
Formosan rock macaque (Taiwan monkey)	*Macaca cyclopis*	NA	40–55*	4–8*	24–31	
Rhesus	*Macaca mulatta*	25	M: 53.2 F: 46.9	M: 7.7 F: 5.34	28	
Cynomolgus	*Macaca fascicularis*	31	M: 41–65 F: 39–50	M: 4.7–8.3 F: 2.5–5.7	28–32 [29, 30]	

NA: not available. *Differences between males and females were not noted.

**Table 2:** Historical NHP PID models and the pathological features found with each subsequent trial.

Researcher, Year	Animal	Chlamydial inoculum	Site of inoculation	Pathologic features
Thygeson and Mengert 1936 [11]	Baboon	Material from infant with inclusion blennorrhea	cervix	Cervicitis, purulent cervical discharge
Alexander et al., 1967 [31]	Taiwan monkey	TRIC	cervix	Mild erythema of the lower genital tract
Ripa et al., 1979 [32]	Grivet	Ct, 2 × 10^5^ IFU/mL	fallopian tube	Histological changes in the upper genital tract, swollen reddened tubes, abundant lymphocytes in the tubal epithelium, clusters of desquamated cells, adhesions between mucosal folds
Johnson et al., 1980 [20]	Marmoset	Ct, 5 × 10^5^ IFU/mL	vagina	Acute inflammatory reaction of the lower genital tract, PMNs, intracytoplasmic chlamydial inclusions
Moller et al., 1980 [33]	Grivet	Ct, 2 × 10^5^ IFU/mL	cervix	Reddened and swollen tubes, exudate from ostia, histological changes in the upper genital tract, lumen of tube diminished and tubal epithelium atrophic and flattened, demonstrated vertical spread of organism
Johnson et al., 1981 [34]	Marmoset	Ct, 4.2–8.8 × 10^5^ IFU/mL	vagina	Demonstrated that reinfection with either homologous or heterologous strain could result in infection however the shorter duration between inoculations resulted in marked immunity and decreased duration of infection
Johnson et al., 1985 [35]	Marmoset	Ct, 5 × 10^5^ IFU/mL	vagina	Cervical erythema with occasional edema, cloudy, or purulent mucus, PMNs identified, endometritis, and salpingitis
Patton et al., 1983, 1984 [36, 37]	Pigtailed macaque	Ct, 6 × 10^6^ IFU/mL	fallopian tube	Acute salpingitis with marked edema and swelling, flocculent exudate, PMNs, isolation of Ct from cervix and tubes
Patton et al., 1987 [38]	Cynomolgus rhesus	Ct, 7 × 10^6^ IFU/mL	Subcutaneous pocket model with fallopian tube implants	Marked erythema, edema, and swelling, widespread inflammation with lymphocytic cells and PMNs, and plasma cells had infiltrated the stroma, infection duration shorter than that seen in intact model with direct tubal inoculation
Patton et al., 1987 [14]	Pigtailed macaque	Ct, 2–4 × 10^8^ IFU/mL	fallopian tube	Chronic salpingitis with extensive tubal scarring, distal tubal obstruction, and peritubal adhesions
Patton et al., 1990 [39]	Pigtailed macaque	Ct, 1 × 10^6^ IFU/mL	cervix	Acute and chronic salpingitis, peritubal adhesions
Wolner-Hanssen et al., 1991 [40]	Pigtailed macaque	Ct, 1 × 10^6^ IFU/mL	cervix	Demonstrated that repeated cervical inoculation resulted protective immunity though there was no relationship between the antibody titer and reinfection
Patton et al., 1994 [41]	Pigtailed macaque	Ct, 5 × 10^3^ IFU/mL	Subcutaneous pocket model with fallopian tube implants	Demonstrated a delayed hypersensitivity in response to inoculation with Ct in both previously infected pockets and noninfected pockets
Van Voorhis et al., 1997 [42]	Pigtailed macaque	Ct, 1 × 10^5^ IFU/mL	Subcutaneous pocket model with fallopian tube implants, cervix and fallopian tubes	Suggests that a Th1-like cytokine response is seen with repeated infection with Ct
Patton et al., 2005 [43]	Pigtailed macaque	Ct, 1 × 10^5^ IFU/mL	cervix	Demonstrated the azithromycin treatment in Ct infection ameliorated the immune response to Ct infection
Patton et al., 2008, 2009 [44, 45]	Pigtailed macaque	Ct, 5 × 10^5^ IFU/mL	vaginal fornix, rectum	Demonstrated the ability of NHP PID model for evaluating the safety and efficacy of topical microbicides
Bell et al., 2010 [21]	Olive baboon	Ct, 1 × 10^7^ IFU/mL	cervix	Acute and chronic salpingitis, peritubal adhesions

Ct: *Chlamydia trachomatis.*
